# Clinical Utility of Machine Learning Methods Using Regression Models for Diagnosing Eosinophilic Chronic Rhinosinusitis

**DOI:** 10.1002/oto2.122

**Published:** 2024-03-10

**Authors:** Hiroatsu Hatsukawa, Masaaki Ishikawa

**Affiliations:** ^1^ Department of Otolaryngology, Head and Neck Surgery Hyogo Prefectural Amagasaki General Medical Center Amgasaki Japan

**Keywords:** asthma, chronic rhinosinusitis, eosinophilia, nasal polyp, regression analysis

## Abstract

**Objective:**

Machine learning methods using regression models can predict actual values of histological eosinophil count from blood eosinophil levels. Therefore, these methods might be useful for diagnosing eosinophilic chronic rhinosinusitis, but their utility still remains unclear. We compared 2 statistical approaches, and investigated the utility of machine learning methods for diagnosing eosinophilic chronic rhinosinusitis.

**Study Design:**

Retrospective study.

**Setting:**

Medical center.

**Methods:**

Data, including eosinophilic levels, obtained from blood and sinonasal samples of 264 patients with chronic rhinosinusitis (257 with and 57 without nasal polyps) were analyzed. We determined factors affecting histopathological eosinophil count in regression models. We also investigated optimal cutoff values for blood eosinophil percentages/absolute eosinophil counts (AECs) through receiver operating characteristic curves and machine‐learning methods based on regression models. A histopathological eosinophil count ≥10/high‐power field was defined as eosinophilic chronic rhinosinusitis.

**Results:**

Blood eosinophil levels, nasal polyp presence, and comorbid asthma were factors affecting histopathological eosinophil count. Cutoffs between the 2 statistical approaches differed in the group with nasal polyps, but not in one without nasal polyps. Machine‐learning methods identified blood eosinophil percentages ≥1% or AEC ≥100/μL as cut‐offs for eosinophilic chronic rhinosinusitis with nasal polyps, while ≥6% or ≥400/μL for one without nasal polyps.

**Conclusion:**

Cut‐offs of blood eosinophil levels obtained by machine‐learning methods might be useful when suspecting eosinophilic chronic rhinosinusitis prior to biopsy because of their ability to adjust covariates, dealing with overfitting, and predicting actual values of histological eosinophil count.

Chronic rhinosinusitis (CRS) is an inflammatory disease of the sinonasal mucosa that adversely affects patients' quality of life.^1^ CRS is subdivided into 2 phenotypes: CRS with and without nasal polyps (CRSwNPs and CRSsNPs, respectively).^2^ Phenotypic CRS studies are ongoing to investigate endotypes. In the inflammatory patterns of sinonasal tissues, CRSwNPs are regarded as “predominantly” type 2 cytokine‐related eosinophilic inflammation, whereas CRSsNPs are considered nontype 2 cytokine‐related inflammation.^3^, ^4^, ^5^ Eosinophilic CRSwNPs involve extensive sinus disease,^6^, ^7^ higher postoperative symptom scores,^7^ and a higher postoperative NP recurrence rate.^8^, ^9^ The European Position Paper on Rhinosinusitis and Nasal Polyps (EPOS) 2020 defined eosinophilic CRS (ECRS) as one of the CRS phenotypes, and ECRS, like CRSwNPs, is considered to be a type 2 cytokine‐related CRS.^2^

Some studies have indicated that eosinophilic CRSsNPs do exist.^7^, ^10^, ^11^, ^12^ Longitudinal studies revealed that CRS with high tissue eosinophilia can be associated with the need for revision surgery independent of the existence of NPs.^10^, ^11^ As type 1‐, 2‐, and 3‐related inflammatory endotypes can be observed in CRSsNPs,^13^ eosinophilic CRSsNPs might be regarded as a type 2 cytokine‐related CRS, similar to eosinophilic CRSwNPs. A recent study reported increased eosinophilic inflammation in the blood and sinonasal samples obtained from patients with type 2 CRSsNPs.^12^ Appropriate diagnosis of eosinophilic CRSsNPs is crucial for predicting treatment outcomes,^11^, ^12^ but it remains unclear which CRSsNP cases should be suspected of having eosinophilic CRSsNPs.

Blood eosinophil percentages <5% and absolute eosinophil count (AEC) <450 to 550/μL is usually considered as normal range, although this depends on laboratory standards.^14^ Blood eosinophilia might be used as an indicator of the severity of eosinophilic inflammation in sinonasal tissues.^10^, ^15^ Eosinophilia is defined by AEC ≥450 to 500/μL, while hypereosinophilia is defined by AEC ≥1500/μL.^14^, ^16^

A histopathological eosinophil count is crucial for ECRS diagnosis. We hypothesized that blood eosinophil levels can be crucial for predicting histopathological eosinophil count in not only CRSwNP but also in CRSsNP. However, it remains unclear whether the eosinophilic association between blood and sinonasal samples differ between CRSwNP and CRSsNP. At present, consensus on the cutoffs for defining ECRS is lacking. Various values of histopathological eosinophil count per high‐power field (HPF; magnification ×400), ranging from 5 to 350, have been established as cutoffs.^17^ The EPOS 2020 defines a histopathological eosinophil count ≥10/HPF as ECRS.^2^ To distinguish ECRS from non‐ECRS, 1 study targeting CRSwNPs defined patients with the percent of histological eosinophils exceeded 10% of total infiltrating cells as ECRS and found the blood eosinophil levels as a factor affecting ECRS diagnosis.^15^ And then, they used a receiver operating characteristic (ROC) curve, and reported the cutoffs of the blood eosinophil percentages and AEC as ≥3.1% and ≥215/μL, respectively.^15^ When applying the regression models, it is possible to predict the actual values of the histological eosinophil count based on the blood eosinophil levels with adjustment of covariates. In addition, a machine learning method can deal with overfitting to data analysis. Therefore, the machine learning method using regression models might be useful for the diagnosis of ECRS, but the utility remains unclear.

In this study, we investigated the utility of the machine learning method using regression models for the diagnosis of ECRS. To this end, we collected data from blood and sinonasal samples previously obtained from patients with CRS and determined factors affecting histopathological eosinophil count by using regression models. Additionally, we adopted 2 statistical approaches to determine the cutoff values of blood eosinophil levels: an ROC curve and the machine‐learning method. And we compared the cutoff values between them.

## Patients and Methods

### Study and Subjects

The research was conducted at Hyogo Prefectural Amagasaki General Medical Center. The Research Ethics Committee of Amagasaki Medical Center approved the study protocol (approval no. 4‐157). Data were obtained retrospectively from patients with CRS (age ≥18 years) who underwent endoscopic sinus surgery in surgical rooms or sinonasal biopsy in outpatient clinics between April 2018 and December 2022. The opt‐out method was used for informed consent. Exclusion criteria were long‐term corticosteroid treatment (>1 month) or use of biologics for systemic inflammatory diseases prior to sinonasal sample collection or diagnosis of anthrochoanal polyps, cystic fibrosis, fungal sinusitis, or acute rhinosinusitis. To eliminate potential short‐term corticosteroid treatment effects on the histopathological eosinophil count, patients who had received oral corticosteroid (OCS) treatment for CRS within 1 month prior to tissue sample collection were excluded.

The following clinical data were collected as explanatory variables for statistical analyses: age, sex, body mass index (BMI), smoking intensity, asthma status, diabetes status, atopy status, allergic rhinitis, anatomic distribution (unilateral/bilateral), Lund‐Mackay computed tomography (CT) scores,^18^ NP presence, blood eosinophil percentage, AEC (/μL), and histopathological eosinophil count. For smoking intensity, the Brinkman index (number of cigarettes smoked per day multiplied by the number of years of smoking) was calculated. Asthma status was categorized as intermittent and persistent asthma. The distinction between intermittent and persistent asthma was necessary to ascertain the status of inhaled corticosteroids (ICS) and OCS use for asthma control at the tissue sampling time point. For comorbid asthma, patients treated with intermittent ICS were referred to as those with intermittent asthma, while the ones treated with ICS but not OCS were referred to as those with persistent asthma. Patients treated with both ICS and OCS were excluded. Diabetes status was categorized as without or with diabetes (currently requiring dietary restrictions or medication). The diagnosis of allergic rhinitis was based on published guidelines.^19^

### Histological Assessment

Sinonasal samples were obtained from NPs in CRSwNP cases and from the mucosa of the ethmoid cavity in CRSsNP cases. The collection of blood and tissue samples did not always occur on the same day. To assess the eosinophilic association between these sample types as accurately as possible, cases with a delay of more than 1 week between sampling sessions were excluded from the analyses. Tissues were fixed in 10% formalin, embedded in paraffin, and cut into thin sections. Hematoxylin‐eosin‐stained sections were used for determining the histopathological eosinophil count. The mean histopathological eosinophil count was calculated using HPF (magnification ×400) microscopy in the 3 densest areas of each slice, with cellular infiltrates beneath the epithelial surface. When the histopathological eosinophil count was ≥10/HPF, patients were defined as having ECRS.^2^

### Data Analysis

Quantitative variables were represented as means or medians with interquartile ranges or 95% confidence intervals (CIs). Age, BMI, smoking intensity, Lund‐Mackay CT scores, blood eosinophil percentages, AEC, and histopathological eosinophil count were defined as continuous variables, whereas sex, diabetes, atopy, allergic rhinitis, NPs, and anatomical distribution were dichotomous variables. Male sex, absence of diabetes/atopy/allergic rhinitis, CRSsNPs, and bilateral distribution were used as reference status. Asthma status was defined as a categorical variable, with no history of asthma used as a reference. For the clinical background comparison between patients with CRSwNPs and CRSsNPs, Mann‐Whitney *U* tests were applied for continuous variables, while Fisher's exact tests were used for dichotomous and categorical variables. Spearman's rank correlation coefficient was used to assess the correlation between blood eosinophil percentages and AEC.

First, we applied univariate and multivariate linear regression models (LRMs) using all data, and then investigated which of the explanatory variables affected histopathological eosinophil count (response variable). As explanatory variables, we introduced blood eosinophil percentages/AEC as well as 10 additional variables (age, sex, BMI, smoking intensity, asthma status, diabetes, atopy, allergic rhinitis, anatomic distribution, and Lund‐Mackay CT scores) into the multivariate LRMs. To narrow down the explanatory variables most relevant to histopathological eosinophil count, we used the least absolute shrinkage and selection operator (LASSO).^20^ In LASSO, the optimal penalty was determined using 10‐fold cross‐validation, whereby *λ* = 0 indicated the usual regression estimates and *λ* = 1 indicated overfitting regression models. To select the variables, the highest penalty with the 1 − standard error rule was applied.^21^

Second, we subdivided patients into those with ECRS and those with non‐ECRS, based on a histopathological eosinophil count ≥10/HPF. We used the ROC curve to calculate the area under the curve (AUC) for CRSwNPs and CRSsNPs separately. We calculated the sensitivity, specificity, and positive‐ and negative‐predictive power/likelihood ratio when using different cutoffs in blood eosinophil percentages/AEC. We defined the cutoff point showing the smallest value for (1 − sensitivity)^2^ + (1 − specificity)^2^ as the optimal cutoff on ROC curves.^22^

Third, using all data, we compared Akaike's information criterion (AIC) between LRMs and generalized additive models (GAMs) to reveal the eosinophilic association between blood and sinonasal samples. While LRMs represent a linear association, the GAM reflects a nonlinear association.^23^ In the GAM formula, blood eosinophil levels/AEC were used to smooth the spline fit.

Fourth, a machine learning method was used to select a better model for predicting histopathological eosinophil count based on blood eosinophil levels. Using the “modelr” package in R (R Foundation for Statistical Computing), data were divided into training and testing data under 10‐fold cross‐validation. We used the training data to introduce the variables most relevant to histopathological eosinophil count selected by LASSO into LRMs and GAMs, and then calculated the AICs. We subsequently calculated the root mean square errors (RMSEs) using the test data. Models with lower AIC and RMSE values were defined as better models to use as the final model for estimating histopathological eosinophil count. A level of 5% was considered statistically significant. R software version 3.6.1 (R Foundation for Statistical Computing) was used for all statistical analyses.

## Results

### Patients' Characteristics

A total of 264 patients were included in this analysis. Patient characteristics are shown in Table 1. In the current study, the proportions of patients with CRS having blood eosinophilia^14^ and hypereosinophilia^16^ were 42% and 13%, respectively. The CRSsNP group had a lower frequency of comorbid asthma, and less eosinophilic inflammation in blood and sinonasal samples than did the CRSwNP group.

**Table 1:** Clinical Characteristics of CRS Patients

	All (N = 264)	CRSwNPs (N = 207)	CRSsNPs (N = 57)	*P* value
Age, y	60 (47, 70)	61 (48, 71)	59 (42, 70)	.31
Male	158 (60%)	133 (64%)	25 (44%)	.01
BMI (kg/m^2^)	23.1 (20.7, 25.7)	23.3 (20.9, 25.7)	22.1 (20.3, 24.7)	.24
Smoking intensity	3 (0, 443)	20 (0, 490)	0 (0, 250)	.26
Asthma status				<.001
No asthma history	142 (54%)	101 (49%)	41 (72%)	
Intermittent asthma	49 (18%)	47 (23%)	2 (3%)	
Persistent asthma	73 (28%)	59 (28%)	14 (25%)	
Diabetes	21 (8%)	17 (8%)	4 (7%)	.99
Atopy	9 (3%)	7 (3%)	2 (4%)	.99
Allergic rhinitis	134 (51%)	118 (57%)	16 (28%)	<.001
Anatomic distribution: Bilateral CRS	239 (91%)	203 (98%)	36 (63%)	<.001
Lund‐Mackay CT score	12 (7, 19)	14 (8, 20)	6 (4, 8)	<.001
Blood eosinophils (%)	7.1 (4.1, 11.7)	7.7 (5.3, 12.0)	3.7 (1.9, 8.3)	<.001
AEC, /μL	433 (250, 807)	465 (316, 818)	211 (100, 444)	<.001
Eosinophilia	110 (42%)	96 (46%)	14 (25%)	.003
Hypereosinophilia	33 (13%)	23 (11%)	10 (18%)	.26
Histopathology eosinophil count (/HPF, ×400)	100 (22, 178)	115 (80, 191)	1 (0, 43)	<.001

Data are shown as number (%) for categorical variables and median (interquartile range) for continuous variables. In comparison between CRSwNPs and CRSsNPs, Fisher's exact tests were applied for categorical variables, while Mann‐Whitney *U* tests were used for continuous variables. Eosinophilia and hypereosinophila represent ≥500 and 1500 of AEC, respectively.

Abbreviations: AEC, absolute eosinophil count; BMI, body mass index; CRS, chronic rhinosinusitis; CRSsNP, CRS without NP; CRSwNPs, CRS with NP; CT, computed tomography; HPF, high‐power field; NP, nasal polyp.

### Selection of Explanatory Variables Affecting Histopathological Eosinophil Count

A log transformation was performed on the histopathological eosinophil count data for the total cohort because the data were positively skewed. The coefficient of correlation between blood eosinophil percentages and AEC was 0.96 (*P* <.0001). These 2 variables were included separately in the regression models as explanatory variables to avoid multicollinearity.

In the univariate LRMs, we observed significant regression coefficients for blood eosinophil percentages, AEC, NP presence, comorbid asthma, allergic rhinitis, anatomic distribution, and Lund‐Mackay CT score (Supplemental Table 1, available online). In the multivariate LRMs, we observed significant regression coefficients for blood eosinophil percentage/AEC, NP presence, comorbid asthma, and anatomical distribution (Table 2). Except for the anatomical distribution, these variables showed positive regression coefficient values, indicating that histopathological eosinophil counts increased with increased blood eosinophil levels, NPs, and comorbid asthma. In the LASSO model, we identified the following explanatory variables as most relevant to histopathological eosinophil count: blood eosinophil percentages/AEC, NPs; and asthma status (Supplemental Figure 1, available online). We introduced these variables as explanatory variables in subsequent analyses.

**Table 2:** Multivariate Linear Regression Models to Evaluate Explanatory Variables Affecting Histopathology Eosinophil Count

	Model using blood eosinophils		Model using AEC	
	Regression coefficient (95% CIs)	*P* value	Regression coefficient (95% CIs)	*P* value
Blood eosinophil percentages	6.0 (4.2, 7.8) × 10^−^^2^	<.001	–	–
AEC	–	–	2.9 (1.8, 4.0) × 10^−^^4^	<.001
Age	0.1 (−1.2, 1.4) × 10^−^^2^	.88	0.1 (−1.2, 1.4) × 10^−^^2^	.86
Female	−1.8 (−6.2, 2.7) × 10^−^^1^	.43	−1.7 (−6.2, 2.9) × 10^−^^1^	.48
BMI	0.0 (−4.4, 4.5) × 10^−^^2^	.98	−0.4 (−5.0, 4.2) × 10^−^^2^	.86
Existence of NP: CRSwNP	2.4 (1.8, 3.0)	<.001	2.5 (1.9, 3.1)	<.001
Smoking intensity	−3.1 (–8.8, 2.5) × 10^−^^4^	.27	−3.2 (−9.0, 2.6) × 10^−^^4^	.28
Asthma status				
Intermittent	0.9 (0.3, 1.4)	<.001	1.0 (0.4, 1.5)	<.001
Persistent	0.8 (0.3, 1.3)	<.001	0.9 (0.5, 1.4)	<.001
With diabetes	−0.1 (−0.8, 0.6)	.80	−0.2 (−0.9, 0.5)	.62
With atopy	0.0 (−1.1, 1,1)	.99	0.1 (−1.0, 1.2)	.88
With allergic rhinitis	−0.9 (−5.0, 3.2) × 10^−^^1^	.58	−0.4 (−4.6, 3.8) × 10^−^^1^	.66
Anatomical distribution: Unilateral CRS	−1.0 (−1.8, –0.2)	<.001	−1.1 (−1.9, –0.3)	<.001
Lund‐Mackay CT score	3.2 (−27.2, 33.7) × 10^−^^3^	.84	0.4 (−30.9, 31.7) × 10^−^^3^	.98

In terms of NP status, CRSsNP was used as a reference. For asthma status, no history of asthma was used as a reference. For anatomic distribution, bilateral CRS was used as a reference.

Abbreviations: AEC, absolute eosinophilic count; BMI, body mass index; CI, confidence interval; CRSsNP, chronic rhinosinusitis without nasal polyp; CRSwNP, chronic rhinosinusitis with nasal polyp; CT, computed tomography.

### Predictive Utility of Blood Eosinophil Levels

We used the ROC curve targeting blood eosinophil percentages/AEC in each of the CRSwNP and CRSsNP groups to calculate the AUC for predicting ECRS (Supplemental Figure 2, available online). In both CRS groups, the AUCs for all blood eosinophil levels exceeded 0.8. These results indicated that the blood eosinophil level is a good predictor of ECRS, independent of whether NPs are present.

The lowest values of (1 − sensitivity)^2^ + (1 − specificity)^2^ were obtained at a blood eosinophil percentage of 5.5% and an AEC of 250/μL in CRSwNPs (Table 3) and a blood eosinophil percentage of 6% and an AEC of 350/μL in CRSsNPs (Table 4).

**Table 3:** Sensitivity, Specificity, Predictive Value, and Likelihood Ratio at Different Cutoff Values of Blood Eosinophil Levels in CRSwNPs

Predictors	Cutoff value	Sensitivity	Specificity	(1 − Sensitivity)^2^ + (1 − Specificity)^2^	Positive‐PV	Negative‐PV	Positive‐LR	Negative‐LR
Blood eosinophil percentage	3	0.93	0.58	0.18	0.96	0.44	2.20	0.13
	3.5	0.90	0.58	0.19	0.96	0.37	2.14	0.18
	4	0.88	0.63	0.15	0.96	0.35	2.40	0.19
	4.5	0.85	0.68	0.12	0.96	0.31	2.68	0.23
	5	0.81	0.68	0.13	0.96	0.27	2.58	0.27
	5.5	0.77	0.79	0.10	0.97	0.25	3.64	0.30
	6	0.74	0.79	0.11	0.97	0.23	3.51	0.33
	6.5	0.68	0.84	0.13	0.98	0.21	4.31	0.38
	7	0.62	0.84	0.17	0.98	0.18	3.91	0.46
AEC, /μL	150	0.95	0.37	0.40	0.94	0.41	1.50	0.14
	200	0.93	0.53	0.23	0.95	0.42	1.95	0.14
	250	0.88	0.68	0.11	0.97	0.36	2.78	0.18
	300	0.80	0.68	0.14	0.96	0.26	2.54	0.29
	350	0.75	0.68	0.16	0.96	0.22	2.38	0.37
	400	0.65	0.84	0.14	0.98	0.20	4.14	0.41
	450	0.56	0.90	0.21	0.98	0.17	5.31	0.49
	500	0.50	0.90	0.26	0.98	0.15	4.75	0.56

Abbreviations: AEC, absolute eosinophil count; CRSwNP, chronic rhinosinusitis with nasal polyp; LR, likelihood ratio; PV, predictive value.

**Table 4:** Sensitivity, Specificity, Predictive Value, and Likelihood Ratio at Different Cutoff Values of Blood Eosinophil Levels in CRSsNPs

Predictors	Cutoff value	Sensitivity	Specificity	(1 − Sensitivity)^2^ + (1 − Specificity)^2^	Positive‐PV	Negative‐PV	Positive‐LR	Negative‐LR
Blood eosinophil percentage	3	0.95	0.58	1.80 × 10^−^^1^	0.53	0.96	2.25	0.09
	4	0.90	0.76	0.67 × 10^−^^1^	0.65	0.94	3.78	0.14
	5	0.84	0.92	0.31 × 10^−^^1^	0.84	0.92	10.67	0.17
	5.5	0.84	0.92	0.31 × 10^−^^1^	0.84	0.92	10.67	0.17
	6	0.84	0.95	0.28 × 10^−^^1^	0.89	0.92	16.00	0.17
	6.5	0.79	0.95	0.47 × 10^−^^1^	0.88	0.90	15.00	0.22
	7	0.79	0.97	0.45 × 10^−^^1^	0.94	0.90	30.00	0.22
AEC, /μL	200	0.95	0.71	0.87 × 10^−^^1^	0.62	0.96	3.27	0.07
	250	0.90	0.84	0.36 × 10^−^^1^	0.74	0.94	5.67	0.13
	300	0.90	0.84	0.36 × 10^−^^1^	0.74	0.94	5.67	0.13
	350	0.90	0.95	0.14 × 10^−^^1^	0.90	0.95	17.00	0.11
	400	0.79	0.95	0.47 × 10^−^^1^	0.88	0.90	15.00	0.22
	500	0.68	0.97	1.01 × 10^−^^1^	0.93	0.86	26.00	0.32

Abbreviations: AEC, absolute eosinophil count; CRSsNP, chronic rhinosinusitis without nasal polyp; LR, likelihood ratio; PV, predictive value.

### Eosinophilic Association Between Blood and Sinonasal Samples Using All Data

**Figure 1:**
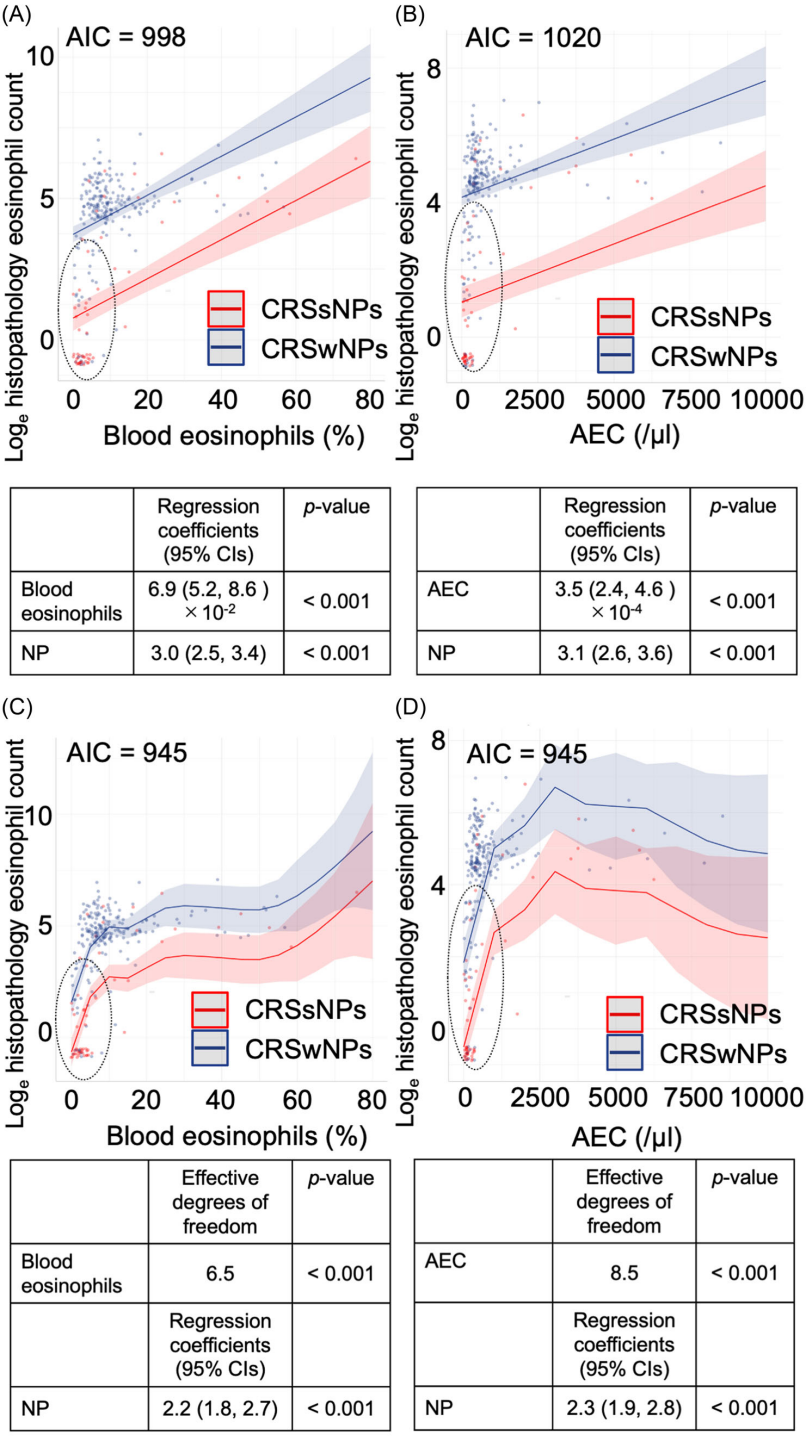
Association between blood eosinophil level and histopathological eosinophil count based on raw data. (A) LRM and blood eosinophils (%), (B) LRM and AEC, (C) GAM and blood eosinophils (%), and (D) GAM and AEC. AEC, absolute eosinophilic count; AIC, Akaike's information criterion; CI, confidence interval; CRSsNP, chronic rhinosinusitis without nasal polyp; CRSwNP, chronic rhinosinusitis with nasal polyp; CT, computed tomography; NP, nasal polyp.

We illustrated the eosinophilic association between blood and sinonasal samples using LRMs (Figure 1A and B). As it was unclear whether the eosinophilic association was linear, we applied the non‐LRM, GAM. In the LRMs targeting blood eosinophil percentages and AEC, the AICs were 998 and 1020, respectively (Figure 1A and B). In the GAMs, both AIC values were 945 (Figure 1C and D). The GAMs showed significant effective degrees of freedom in blood eosinophil levels and regression coefficients for the presence of NPs. The AIC findings indicated that GAMs may be more accurate models than LRMs for predicting histopathological eosinophil counts. We observed the inaccuracy with which LRMs were able to predict some data (blood eosinophil percentage <10% and AEC <500/μL) (see the dotted circles in Figure 1A and B). These data could be predicted by applying GAMs (dotted circles in Figure 1C and D).

### Selection of Final Models to Predict Histopathological Eosinophil Count Using a Machine Learning Method

Predictive models using all available data are prone to overfitting. Additionally, whether the interaction between blood eosinophil levels and NPs affects histopathological eosinophil count prediction was unclear. To overcome these issues, we prepared the following 4 models and used the machine‐learning method: LRMs and GAMs with/without an interaction term (blood eosinophil percentage/AEC × NPs). In both models, targeting blood eosinophil percentages and AEC, GAMs with an interaction term showed the lowest AICs and RMSEs values (Supplemental Table 2, available online), indicating that these models performed better. Therefore, we defined GAMs with an interaction term as the final model for predicting histopathological eosinophil count. In the final model, we observed significant interaction effects: the predicted regression coefficient (95% CIs) for blood eosinophil percentages × NPs was −8.2 (−8.5, −8.0) × 10^−2^ (*P* <.0001), while that for AEC × NPs −7.2 (−7.4, −7.0) × 10^−4^ (*P* <.0001). The significant negative values indicated a narrower difference in histopathological eosinophil count between CRSwNPs and CRSsNPs due to increased blood eosinophil levels. The findings were remarkable when blood eosinophil percentages >10% and AEC >1000/μL.

### Prediction of Histopathological Eosinophil Count Using Final Models

**Figure 2:**
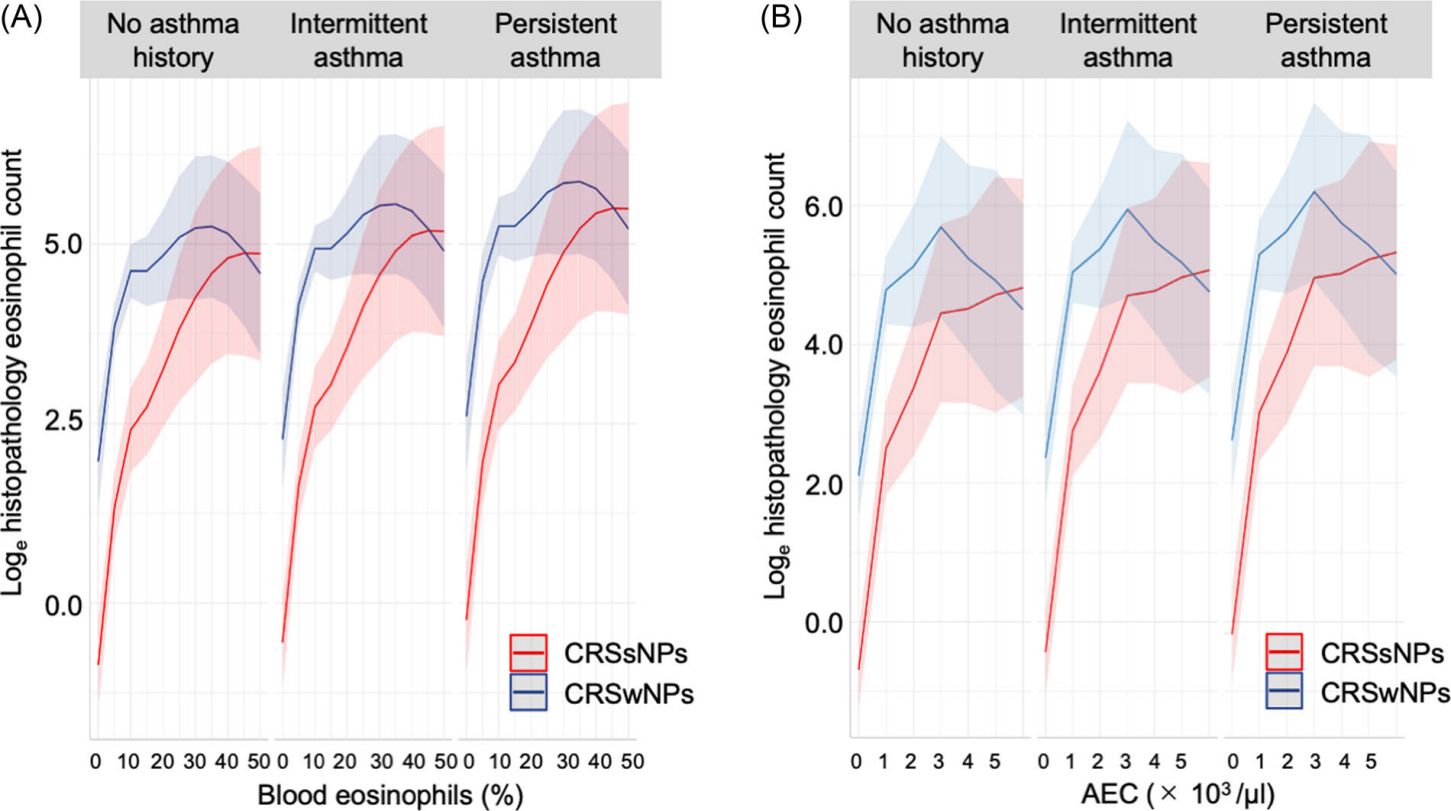
Final models for predicting log‐transformed histopathological eosinophil count. GAM prediction using blood eosinophils (A) and AEC (B). AEC, absolute eosinophilic count; CRSsNP, chronic rhinosinusitis without nasal polyp; CRSwNP, chronic rhinosinusitis with nasal polyp.

To visualize the association between the eosinophil levels in blood and sinonasal samples, we plotted the final model results using log‐transformed histopathological eosinophil count (Figure 2). The slopes for CRSwNPs and CRSsNPs were identical when eosinophil percentages were <10% and AEC was <1000/μL. Above these values, the slope was more gradual for CRSwNPs than for CRSsNPs.

**Figure 3:**
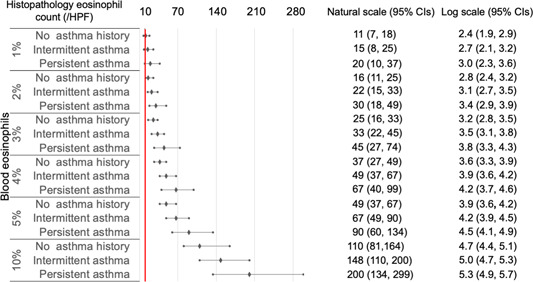
Histopathological eosinophil count predicted by final models using blood eosinophil percentages in chronic rhinosinusitis with nasal polyps. All values represent means and 95% confidence intervals (CIs). A red line indicates 10/high‐power field (HPF) in terms of histopathological eosinophil count.

**Figure 4:**
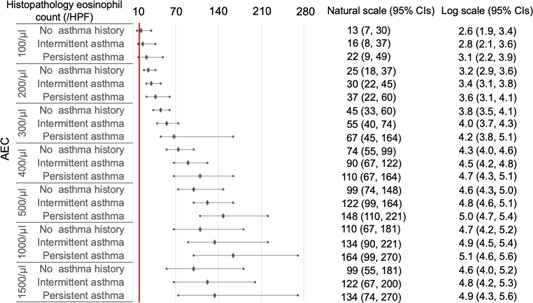
Histopathological eosinophil count predicted by final models using absolute eosinophilic count (AEC) in chronic rhinosinusitis with nasal polyps. All values represent means and 95% confidence intervals (CIs). A red line indicates 10/high‐power field (HPF) in the histopathological eosinophil count.

As histopathological eosinophil count on natural scales may be more familiar to clinicians than that on log‐transformed scales, we transformed the log scales into natural scales for subsequent analyses. In CRSwNP cases with blood eosinophil percentages ≥1% and AEC ≥100/μL (Figures 3 and 4), the 95% CIs of predicted histopathological eosinophil count were included or exceeded 10/HPF, independent of asthma status.

**Figure 5:**
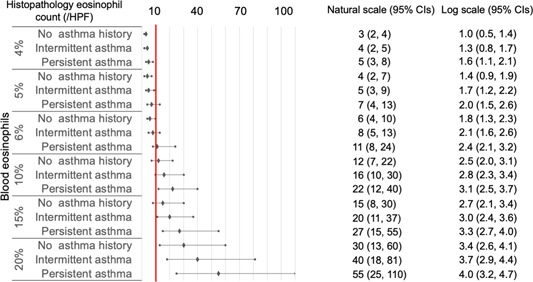
Histopathological eosinophil count predicted by final models using blood eosinophil percentages in chronic rhinosinusitis without nasal polyps. All values represent means and 95% confidence intervals (CIs). A red line indicates 10/high‐power field (HPF) in terms of the histopathological eosinophil count.

**Figure 6:**
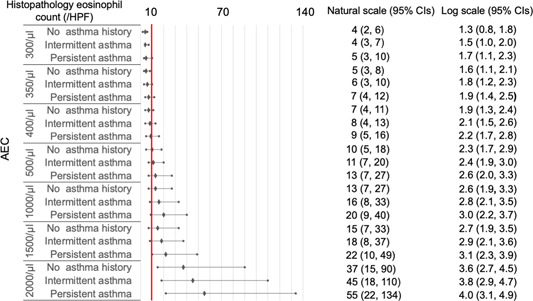
Histopathological eosinophil count predicted by final models using absolute eosinophilic count (AEC) in chronic rhinosinusitis without nasal polyps. All values represent means and 95% confidence intervals (CIs). A red line indicates 10/high‐power field (HPF) in terms of the histopathological eosinophil count.

In CRSsNP cases with blood eosinophil percentages ≥6% and AEC ≥400/μL (Figures 5 and 6), the 95% CIs of the predicted histopathological eosinophil count were included or exceeded 10/HPF, independent of asthma status.

## Discussion

In this study, we used 2 approaches to determine blood eosinophil thresholds for predicting eosinophilic forms of CRSwNPs and CRSsNPs. The ROC curve method showed that the optimal cutoffs were 5.5% of blood eosinophil percentages and 250/μL of AEC in CRSwNPs, while those for CRSsNPs were 6% and 350/μL, respectively. A nonlinear association was observed between blood and sinonasal sample eosinophil levels. The machine‐learning methods showed that the optimal cutoffs were a blood eosinophil percentage of 1% and an AEC of 100/μL for CRSwNPs, while those for CRSsNPs were 6% and 400/μL, independent to asthma status.

For CRSsNPs, the anatomical location for obtaining sinonasal samples for histopathological eosinophil count can be critical. In one study investigating endotypes of CRSsNPs among the inferior turbinate, uncinate, and ethmoid tissues, the most severe and highest inflammation heterogeneity was observed in the ethmoid tissues.^24^ In our study, all CRSsNPs samples were taken from ethmoid tissues. It remains unclear whether tissue sampling from the inferior turbinate and uncinate tissues would lead to the same interpretation.

When we compared the cutoff values of blood eosinophil percentages/AEC, the 2 approaches yielded different results for CRSwNPs. In the ROC curve, the data of CRSwNPs and CRSsNPs were analyzed separately, and the cutoffs were calculated without accounting for the effects of asthma status as the covariate. In the machine‐learning method, the data of CRSwNPs and CRSsNPs were analyzed together, and the cutoffs were calculated by adjusting for the effects of NPs and asthma status as covariates. In particular, a higher ratio of comorbid asthma was observed in CRSwNPs (see Table 1). The different cutoff values observed in CRSwNPs could be attributed to the presence or absence of adjusting effects of asthma status.

To date, no consensus for cut‐offs of histological eosinophil count has been established for defining ECRS.^17^ Therefore, the machine learning methods using GAMs might be more practical than ROC curves for diagnosing ECRS, due to their ability of adjusting covariates, dealing with the issue of overfitting, and predicting actual values of the histological eosinophil count. In addition, our data can be applied according to clinicians' preferences for cut‐off values of histological eosinophil count for defining ECRS. For instance, ECRS is defined as HPE ≥70/HPF in Japan.^25^ ECRS can be predicted in CRSsNPs cases showing ≥20% blood eosinophil percentages with comorbid asthma and AEC ≥2000/μL independent of asthma status. In particular, the prediction of eosinophilic CRSsNPs prior to biopsy might help physicians realize the possibility of poor treatment outcomes, despite the absence of NPs.

Our study had several limitations. First, other explanatory variables—such as use of intranasal corticosteroid, or the dose of ICS treatment for comorbid asthma—that were not applied in the present study (due to the retrospective nature of the study) might have affected the outcome. Our data did not show dose‐dependent effects of ICS on histopathological eosinophil count. Further studies are necessary to investigate the dose‐dependent effects of ICS on histological eosinophil count. Second, the small sample sizes—particularly in CRS cases with blood eosinophil percentages >10% and AEC >1000/μL—may have limited the prediction of histopathological eosinophil count. Higher blood eosinophil counts resulted in a wider range of 95% CIs, which clinicians should be aware of.

## Conclusion

The cut‐off values of blood eosinophil levels obtained using the machine‐learning method developed in this study might be useful for predicting ECRS prior to biopsy. In particular, the early prediction of eosinophilic CRSsNPs might be useful for explaining to the patients about the possibility of poor treatment outcomes, despite the absence of NPs.

## Author Contributions

**Hiroatsu Hatsukawa**, drafted the manuscript and provided administrative, technical, and material support, read and approved the final version of the manuscript for submission; **Masaaki Ishikawa**, concept and design of the study, revision of the manuscript, and statistical analyses, drafted the manuscript and provided administrative, technical, and material support, read and approved the final version of the manuscript for submission.

## Disclosures

### Competing interests

None.

### Funding source

This work was supported by JSPS KAKENHI Grant Number 21K16850.

## Supporting information

Supporting information.

Supporting information.

Supporting information.

## Data Availability

Data supporting the findings of this study are available upon request from the corresponding authors.
