# Supplementary material for: Clinical Utility of Machine Learning Methods Using Regression Models for Diagnosing Eosinophilic Chronic Rhinosinusitis
Source: OTO Open. 2024 Mar 10;8(1):e122. doi: 10.1002/oto2.122 (PMC10924764; doi:10.1002/oto2.122)
Supplement: Supplementary file 1 — Supporting information. [file OTO2-8-e122-s003.docx]

**Supplementary Figure 1**. **Cross-validation plot for selection of explanatory variables relevant to the histopathological eosinophil count.**

Top rows represent the number of non-zero coefficients per penalty value; dots indicate the binomial deviance. The left vertical lines indicate optimal penalty and right vertical lines indicate the largest penalty values related to the 1-SE rules. (a) Model using blood eosinophil percentages, and (b) model using AEC. The tables show the clinical variables and coefficients selected by the 1-SE rule. Blood eosinophil percentages/AEC, asthma status, and existence of NP were selected as explanatory variables most relevant to histopathological eosinophil count.

Abbreviations: AEC, absolute eosinophil count; CRSwNPs, chronic rhinosinusitis with nasal polyps; SE, standard error; NPs, nasal polyps.
