# Supplementary material for: Clinical Utility of Machine Learning Methods Using Regression Models for Diagnosing Eosinophilic Chronic Rhinosinusitis
Source: OTO Open. 2024 Mar 10;8(1):e122. doi: 10.1002/oto2.122 (PMC10924764; doi:10.1002/oto2.122)
Supplement: Supplementary file 2 — Supporting information. [file OTO2-8-e122-s002.docx]

**
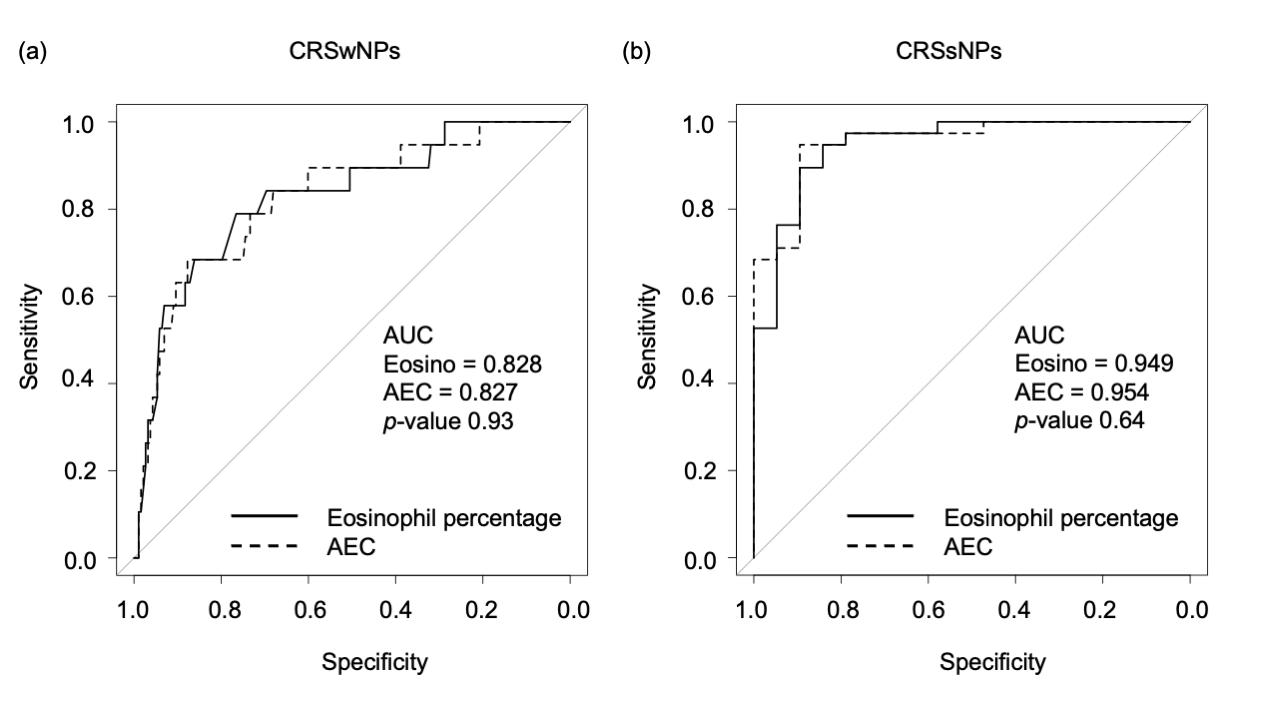
Supplementary Figure 2**. Comparison of AUCs between the blood eosinophil percentage and AEC in ROC curves. (a) CRSwNPs, and (b) CRSsNPs.

Abbreviations: AEC, absolute eosinophil count; AUC, area under the curve; CRSwNPs, chronic rhinosinusitis with nasal polyps; CRSsNPs, chronic rhinosinusitis without nasal polyps; ROC, receiver operating characteristic.
