# Supplementary material for: Clinical Utility of Machine Learning Methods Using Regression Models for Diagnosing Eosinophilic Chronic Rhinosinusitis
Source: OTO Open. 2024 Mar 10;8(1):e122. doi: 10.1002/oto2.122 (PMC10924764; doi:10.1002/oto2.122)
Supplement: Supplementary file 3 — Supporting information. [file OTO2-8-e122-s001.docx]

| **Supplementary Table 1. Results of univariate linear regression models for evaluating explanatory variables affecting histopathological eosinophil count** | | | | |
| --- | --- | --- | --- | --- |
|  | Regression coefficient (95% CIs) | | | *p*-value |
| Blood eosinophil percentages | 7.2 (5.1, 9.3) ×10^–2^ | | | < 0.001 |
| AEC | 2.9 (1.6, 4.3) ×10^–4^ | | | < 0.001 |
| Age | 0.2 (–1.5, 1.9) ×10^–2^ | | | 0.80 |
| Female | –1.7 (–7.0, 3.6) ×10^–1^ | | | 0.53 |
| BMI | –2.0 (–8.1, 4.1) ×10^–2^ | | | 0.52 |
| Existence of NP: CRSwNPs | 3.0 (2.5, 3.5) | | | < 0.001 |
| Smoking intensity | –0.7 (–7.7, 6.3) ×10^–4^ | | | 0.85 |
| Asthma status |  |  |  |  |
| Intermittent asthma | 1.7 (1.0, 2.3) | | | < 0.001 |
| Persistent asthma | 1.5 (0.9, 2.0) | | | < 0.001 |
| With diabetes | –0.5 (–1.4, 0.5) | | | 0.36 |
| With atopy | 0.1 (–1.4, 1,5) | | | 0.93 |
| With allergic rhinitis | 0.7 (0.2, 1.2) | | | < 0.001 |
| Anatomic distribution:  Unilateral CRS | –3.4 (–4.2, –2.6) | | | < 0.001 |
| Lund–Mackay CT score | 0.8 (0.4, 1.1) ×10^–1^ | | | < 0.001 |
| For existence of NP, CRSsNPs was used as a reference. For asthma status, no history of asthma was used as a reference. For anatomic distribution, bilateral CRS was used as a reference.  Abbreviations: AEC, absolute eosinophil count; BMI, body mass index; CIs, confidence intervals; CRSsNPs and CRSwNPs, chronic rhinosinusitis without and with nasal polyps, respectively; CT, computed tomography. | | | | |

| **Supplementary Table 2. Comparison of AIC and RMSE among four models** | | | | |
| --- | --- | --- | --- | --- |
| Main explanatory variables | Blood eosinophil percentages | | AEC | |
|  | AIC | RMSE | AIC | RMSE |
| LRM | 893 | 47.2 | 907 | 49.4 |
| LRM with an interaction term | 883 | 40.2 | 904 | 47.1 |
| GAM | 852 | 37.0 | 855 | 37.2 |
| GAM with an interaction term | 844 | 30.3 | 849 | 32.4 |
| LRM: Log_e_ histopathology eosinophil count = intercept + blood eosinophil percentages or AEC + NP + Asthma  GAM: Log_e_ histopathology eosinophil count = intercept + s (blood eosinophil percentages or AEC) + NP + Asthma  [The function “s” is used for smoothing the spline fit.]  Interaction term: blood eosinophil percentages or AEC × NP  Abbreviations: AEC, absolute eosinophil count; AIC, Akaike’s information criterion; GAM, generalized additive model; LRM, linear regression model; RMSE, root mean squared error | | | | |
